# Efficacy of fermented foods in irritable bowel syndrome: a systematic review and meta-analysis of randomized controlled trials

**DOI:** 10.3389/fnut.2024.1494118

**Published:** 2025-01-07

**Authors:** Liang Ding, Jinnan Duan, Tao Yang, Mengping Yuan, A. Huo Ma, Yuehua Qin

**Affiliations:** ^1^Department of Gastroenterology, Shaoxing People’s Hospital, Shaoxing, China; ^2^Department of Infectious Diseases, Shaoxing People’s Hospital, Shaoxing, China

**Keywords:** fermented foods, irritable bowel syndrome, probiotics, efficacy, meta-analysis

## Abstract

**Objective:**

Fermented foods (FFs) may theoretically benefit irritable bowel syndrome (IBS) symptoms, but the role of FFs for IBS patients in the real world is inconsistent and has not been systematically assessed. We performed a systematic review and meta-analysis to examine this issue.

**Methods:**

PubMed, MEDLINE, Embase and Cochrane Library databases were searched up to August 2024. Randomized controlled trials (RCTs) investigating the efficacy of FFs in IBS were eligible for the analysis. Two authors independently screened studies and extracted data. Data were pooled using relative risk (RR) of dichotomous data and standardized mean difference (SMD) for continuous data.

**Results:**

A total of 16 RCTs with 1,264 IBS patients were included. There were 12 RCTs involving 975 patients providing primary outcomes which was defined as symptom relief. The proportion of symptom relief was associated with the administration of FFs (RR 1.22, 95% CI 1.04–1.42, *p* = 0.01, *I*^2^ = 0%). For secondary outcomes, FFs also exerted a beneficial effect on global symptoms scores (SMD = −0.15; 95% CI −0.29 to −0.02, *p* = 0.02, *I*^2^ = 46%), but no significant improvement on abdominal pain scores and bloating scores. Subgroup analysis showed that fermented milk had a beneficial effect on symptom relief (RR 1.19, 95% CI 1.01 to 1.39, *p* = 0.04, *I*^2^ = 0%).

**Conclusion:**

Fermented foods, especially fermented milk with probiotics properties, appear to be efficacious in irritable bowel syndrome. However, given the limitations of current evidence, this conclusion should be interpreted with caution.

**Systematic Review Registration:**

This study was registered on the International Prospective Register of Systematic Reviews (PROSPERO) as CRD42024576608.

## 1 Introduction

Irritable bowel syndrome (IBS) is a functional gastrointestinal disorder characterized by symptoms of recurrent abdominal pain related to disordered bowel habits (1). The troublesome condition is associated with mental disorders, impaired life quality, and reduced social functioning, for example, 1/4 patients report sickness-related absences from work (2). In addition, it remains one of the most common diseases, with a global prevalence of 3.8–10.1% in the general population according to the data from a large-scale global survey by Rome Foundation (3) and an updated meta-analysis (4). Thus, IBS causes a substantial social healthcare burden from direct or indirect influence, estimated to be in excess of £1 billion in the United Kingdom (5), €3–4 billion in Germany (6), $2 billion in China (7) and $9 billion in the USA (8) per year.

Although the etiology of IBS remains incompletely understood, there is a consensus that diet plays a vital role in the management of IBS. Over 80% of patients reported food-related symptoms, and current guidelines and dietetic associations supported that dietary therapy is effective in managing IBS (9–11). In addition, a series of high-quality meta-analyses of RCTs indicated that probiotics are deemed beneficial for IBS by comprehensively considering the effect, cost and safety (12, 13).

Fermented foods (FFs), defined as “foods made through desired microbial growth and enzymatic conversions of food components” by The International Scientific Association for Probiotics and Prebiotics, are popular among nearly every nation worldwide due to their unique textures, flavors and biological functionalities (14). Over the past decade, the popularity of FFs has emerged strongly, partially attributed to a renewed appreciation of their health benefits, supported by evidence from omics-based technologies (15). Interestingly, the evidence confirmed that the fermentation process has the potential to increase probiotic/prebiotic content and reduce undesirable compounds, such as FODMAPs (fermentable oligosaccharides, disaccharides, monosaccharides and polyols), which is believed to be beneficial for IBS in theory (16, 17). However, the role of FFs for IBS patients in the real world is inconsistent and has not been systematically assessed. Therefore, we aimed to perform a Systematic Review and Meta-analysis of randomized controlled trials (RCTs) to estimate the efficacy of FFs in IBS.

## 2 Methods

This study was registered on the International Prospective Register of Systematic Reviews (PROSPERO) as CRD42024576608, and reported according to the Preferred Reporting Items for Systematic Reviews and Meta-Analyses (PRISMA) recommendations (18).

### 2.1 Search strategy

We searched PubMed, MEDLINE, Embase and Cochrane Library databases up to August 2024, using combinations of the keywords: “irritable bowel syndrome” and “fermented food.” No language restrictions were applied. In addition, the reference lists of all identified relevant studies or reviews were adopted to perform a manual search of the literature. The specific search strategy to identify the studies about the correlation between fermented foods and IBS was reported in Supplementary material 1. The literature management was conducted in EndNote 21 (Clarivate Analytics, US).

### 2.2 Inclusion and exclusion criteria

PICOS (Patient, Intervention, Comparators, Outcome, and Study design) inclusion criteria were developed in our study.

Inclusion criteria: (1) P: IBS patients diagnosed by Rome or Manning criteria, no restrictions on age, race, or sex. (2) I: The treatment group received fermented food in any form. (3) C: The control group received an appropriate placebo that should contain similar ingredients but without extra fermented process. (4) O: Studies reported dichotomous or continuous data on the clinical effect of IBS symptoms. (5) S: The clinical trials conformed to the random allocation and controlled principles. Of note, crossover studies with an adequate washout period were also considered eligible.

Exclusion criteria: (1) studies not adhering to the inclusion criteria, (2) studies without eligible data for any reasons, (3) studies that include other interventions like drug, acupuncture, yoga or herb, and (4) experimental trials on animals or non-human studies.

### 2.3 Outcome assessment

The primary outcome was dichotomous data that included either symptom cure or improvement which reflect the effect of fermented food compared with placebo on global IBS symptoms. The secondary outcomes were continuous data that included the symptom scores of global IBS symptoms, abdominal pain and abdominal bloating.

Adverse events (AEs) were also recorded to assess the safety.

### 2.4 Data extraction

Two reviewers (LD and JND) extracted the target data independently onto a standardized spreadsheet. Discrepancies and disagreements were resolved by discussion and an additional reviewer (YHQ). Data extraction included the items of RCT general information, population characteristics, and outcomes of interest. Where a study failed to provide sufficient data, corresponding authors were queried for original information by e-mail. If necessary, we used Plot Digitizer software to estimate the target data from statistical chart according to the recommendation of the Cochrane Handbook (19). Intention-to-treat analyses (ITT) were performed and withdrawal or loss to follow-up were assumed to be treatment failures.

### 2.5 Quality assessment and risk of bias

Similarly, LD and JND evaluated the quality of included studies, and disagreements were resolved by discussion with a senior investigator (YHQ). Firstly, we assessed the study quality using the Jadad scale, which records whether a study is fully described to random sequence production, blind method and withdrawal. Scores ≥3 were deemed to be high quality. Then, we generated a risk of bias graph (including selection bias, performance bias, detection bias, attrition bias, reporting bias and other bias) by Review Manager 5.3 (The Cochrane Collaboration, Oxford, United Kingdom) following the instructions of Cochrane Handbook (20).

### 2.6 Statistical analyses

The estimated effects of dichotomous data were synthesized by risk ratio (RR), and continuous data were synthesized by standardized mean difference (SMD). Inter study heterogeneity was measured by the chi-square-based *Q* statistical test and quantified by *I*^2^ statistic to evaluate the true intervention effect in different studies. *I*^2^ ≥ 50% was deemed to represent significant heterogeneity, and the estimated effects were measured by a random-effect model. On the contrary, if statistical heterogeneity was not observed (*I*^2^ < 50%), a fixed effects model was used. The sensitivity analysis was conducted to verify the outcome robustness by using the single study deletion method. Funnel plot, Begg adjust rank correlation and Egger regression asymmetry test were performed to examine the possibility of publication bias. In addition, we performed subgroup analyses based on fermented food type.

All statistical analyses were conducted using Review Manager 5.3, except the Begg and Egger tests, which were performed using Stata SE 15 (StataCorp, College Station, TX).

## 3 Results

**Figure 1:**
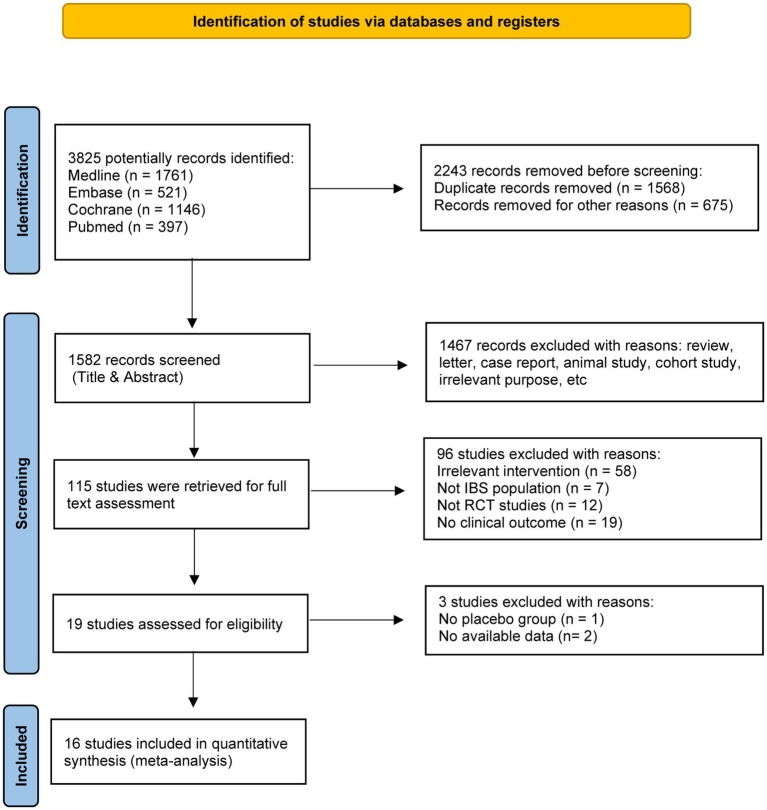
Flow diagram of literature search and selection.

A total of 3,825 records were identified in the initial computerized search, of which 115 published studies appeared to be relevant and were retrieved for full-text appraisal. Of these, 96 studies were excluded for various reasons, leaving 16 eligible studies for further qualitative synthesis (Figure 1). These 16 trials involved 1,264 IBS patients including 1,008 female, and the proportion of female was highly to 79.7% (21–36). There were 8 trials used fermented milk, 3 fermented oat, 2 fermented wheat flours, 1 fermented rice, 1 kombucha, and 1 sauerkraut. All the studies were randomized controlled trials. Of these, 13 studies were double-blind trials, 2 studies were single-blind trials (22, 36), and one was an open-label trial (24). In addition, most of the studies followed a parallel-group design, but the studies of Laatikainen et al. (27) were crossover RCTs. More information was summarized in Table 1.

**Table 1:** Basic characteristics of the included studies.

Author	Year	Region	Criteria	Duration	Fermented foods	IBS Subtype	Intervention group		Control group		Jadad score
							*N* (Female)	Age (years) (mean ± SD)	*N* (Female)	Age (years) (mean ± SD)	
Agrawal et al. (21)	2008	UK	Rome III	4 weeks	Fermented milk	IBS-C	17 (17)	42 (24, 69)^a^	17 (17)	37 (20, 59)^a^	3
Bednarska et al. (22)	2022	Sweden	Rome IV	2 weeks	Fermented oat	IBS-D/M	18 (16)	37 (19, 55)^a^	12 (9)	37 (19, 55)^a^	2
Guyonnet et al. (23)	2007	France	Rome II	6 weeks	Fermented milk	IBS-C	135 (106)	49.4 ± 11.4	132 (93)	49.2 ± 11.4	4
Isakov et al. (24)	2023	Russia	Rome IV	10 days	Kombucha	IBS-C	20 (20)	48.7 ± 17.7	20 (20)	47.7 ± 15.9	3
Kajander et al. (25)	2008	Finland	Rome II	20 weeks	Fermented milk	IBS-D/C/M	43 (41)	50 ± 13	43 (39)	46 ± 13	5
Kim et al. (26)	2024	Korea	Rome IV	4 weeks	Fermented rice drink	IBS-D/C/M	30 (24)	39.17 ± 12.8	30 (25)	37.40 ± 8.30	2
Laatikainen et al. (27)	2016	Finland	Rome III	4 weeks	Sourdough bread	IBS-D/U/M	80 (73)	42.9 (21, 64)^a^	80 (73)	42.9 (21, 64)^a^	4
Lluansí et al. (28)	2024	Spain	Rome IV	8 weeks	Sourdough bread	IBS-D/C/M/U	12 (7)	50.56 ± 11.65	11 (6)	45.40 ± 12.88	5
Niedzielin et al. (29)	2001	Poland	Manning criteria	4 weeks	Fermented oat	IBS-D/C/M	20 (15)	45 ± 18	20 (17)	42 ± 15	3
Nielsen et al. (30)	2018	Norwegian	Rome III	6 weeks	Sauerkraut	IBS-D/C/M/U	27 (24)	42.0 ± 10.2	31 (25)	38.2 ± 13.4	5
Nobaek et al. (31)	2000	Sweden	Rome I	4 weeks	Fermented oat	Not stated	25 (16)	51 (24, 78)^a^	27 (20)	46 (21, 66)^a^	3
Roberts et al. (32)	2013	UK	Rome III	12 weeks	Fermented milk	IBS-C/M	88 (73)	44.6 ± 11.98	91 (76)	43.71 ± 12.76	5
Simrén et al. (33)	2010	Sweden	Rome II	8 weeks	Fermented milk	IBS-D/C/M	37 (26)	42 ± 15	37 (26)	44 ± 16	5
Søndergaard et al. (34)	2011	Denmark	Rome II	8 weeks	Fermented milk	Not stated	27 (20)	53.9 (29, 67)^a^	25 (19)	48.5 (29, 67)^a^	5
Thijssen et al. (35)	2016	Netherlands	Rome II	8 weeks	Fermented milk	IBS-D/C/M/U	39 (26)	41.1 ± 14.8	41 (29)	42.4 ± 13.5	4
Zeng et al. (36)	2008	China	Rome II	4 weeks	Fermented milk	IBS-D	14 (4)	44.6 ± 12.4	15 (6)	45.8 ± 9.2	2

IBS, irritable bowel syndrome; C, constipation; D, diarrhea; U, unclassified; M, mixture of diarrhea and constipation; N, number of patients.

^a^Age (years) (mean, range).

### 3.1 Study quality and risk of bias

The quality of the studies was generally good, with 9 (56.3%) scoring ≥4 on the Jadad scale (Table 1). The risk-of-bias analysis also indicated a low or unclear risk of bias regarding selection bias. However, a high risk of bias was frequently observed in attrition bias and reporting bias. Details were presented in Supplementary material 2.

### 3.2 Meta-analysis

#### 3.2.1 Effect on symptom relief

**Figure 2:**
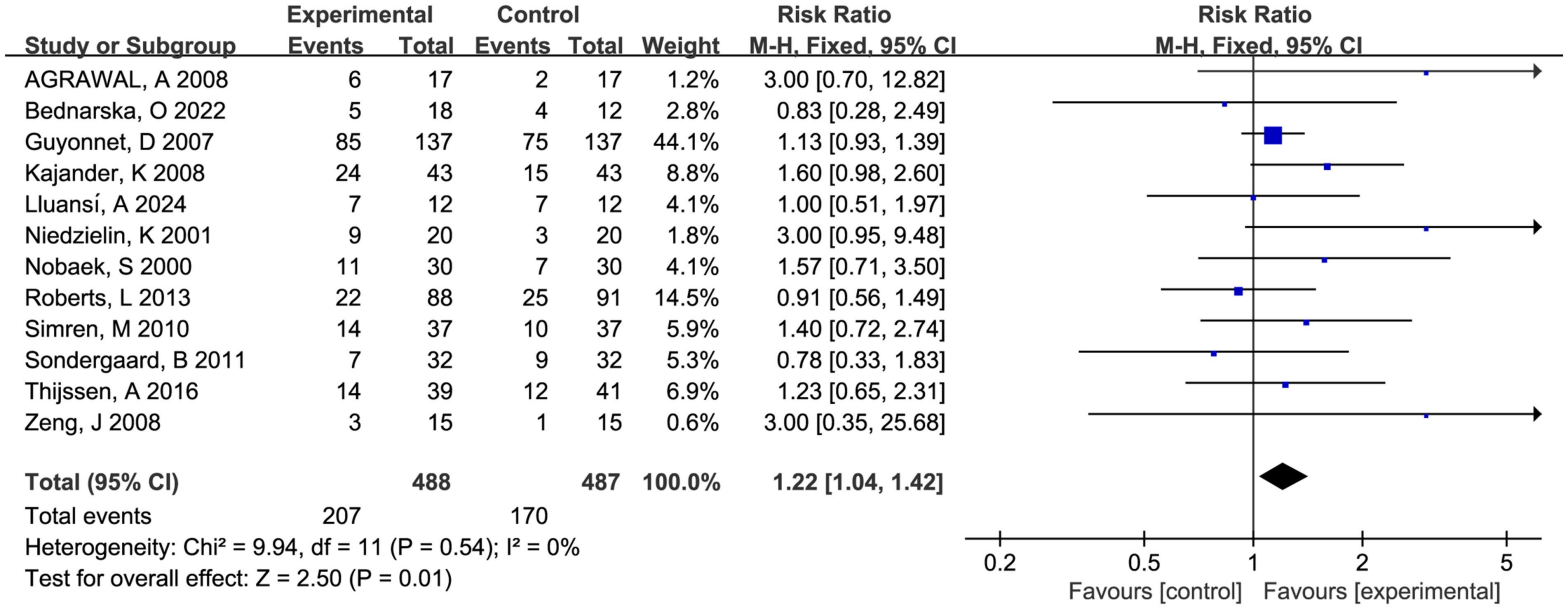
Forest plot of comparison between fermented foods and placebo in term of symptom relief. Experimental: fermented food group. Control: placebo group.

Twelve RCTs compared fermented foods with placebo in term of symptom relief. Overall, 207 (42.4%) of 488 patients assigned to the experimental group reported symptom relief from IBS symptoms following therapy, compared with 170 (34.9%) of 487 allocated to control. Fermented foods had a statistically significant effect in improving IBS symptoms (RR 1.22, 95% CI 1.04–1.42, *p* = 0.01, *I*^2^ = 0%) (Figure 2).

Sensitivity analysis confirmed the good heterogeneity and outcome robustness through single study deletion (Supplementary material 5). No significant funnel plot asymmetry was observed (*P* > 0.1 in the Begg and Egger test, Supplementary materials 3, 4).

#### 3.2.2 Effect on symptom score

**Figure 3:**
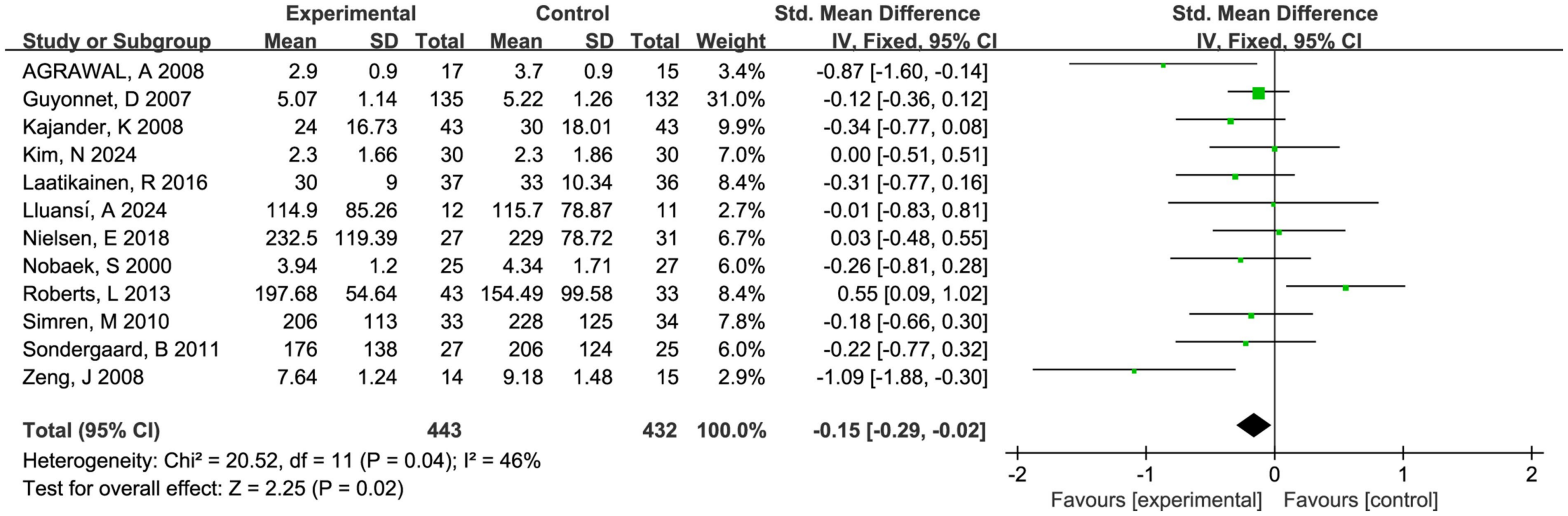
Forest plot of comparison between fermented foods and placebo in term of global symptom score. Experimental: fermented food group. Control: placebo group.

Twelve trials involving 875 patients reported global symptom scores. There was a statistically significant benefit in favor of fermented foods improving global symptom score (SMD = −0.15; 95% CI −0.29 to −0.02, *p* = 0.02, *I*^2^ = 46%) (Figure 3). Sensitivity analysis confirmed the acceptable heterogeneity through single study deletion, but the outcome robustness was inconsistent in the process (Supplementary material 6). No significant publication bias was observed in the funnel plot and asymmetry test (*P* > 0.1 in Begg and Egger test, Supplementary materials 3, 4).

**Figure 4:**
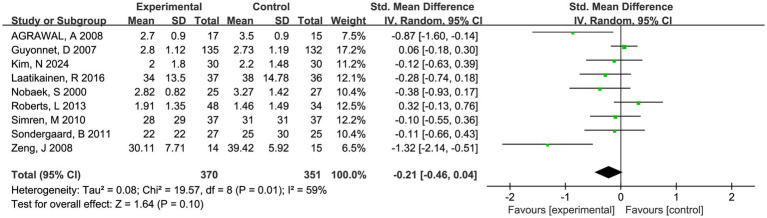
Forest plot of comparison between fermented foods and placebo in term of abdominal pain score. Experimental: fermented food group. Control: placebo group.

**Figure 5:**
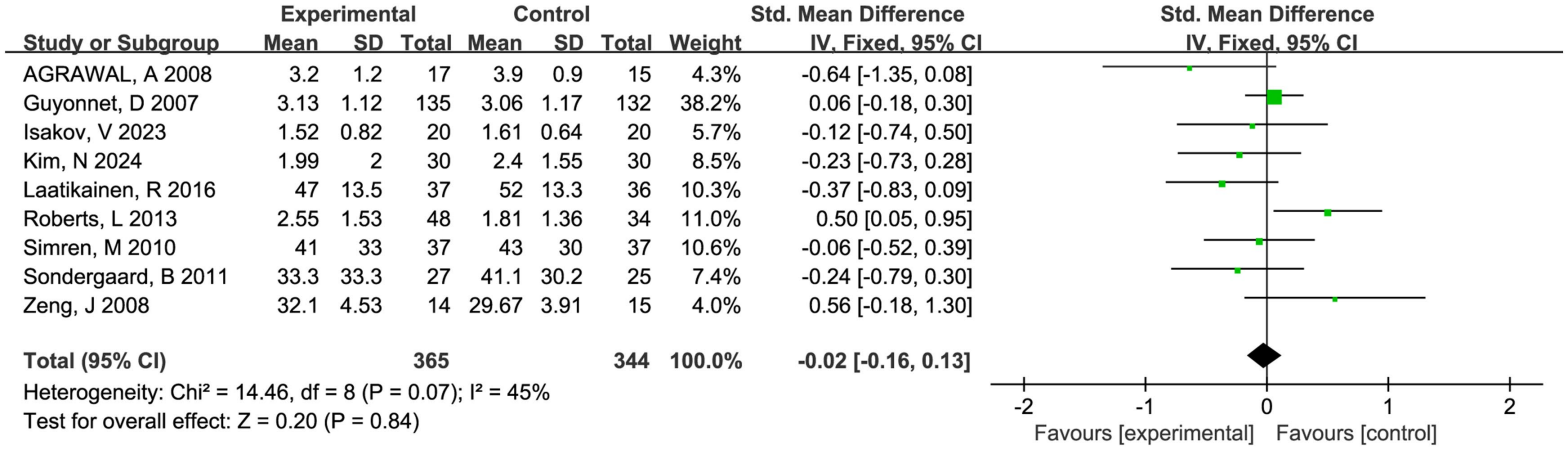
Forest plot of comparison between fermented foods and placebo in term of abdominal bloating score. Experimental: fermented food group. Control: placebo group.

Nine trials involving 721 patients reported abdominal pain scores and nine trials involving 709 patients reported abdominal bloating scores, respectively. There was a trend toward fermented foods improving abdominal pain and bloating, but the statistical outcomes were insignificant (*P* > 0.05, Figures 4, 5).

#### 3.2.3 Adverse events

**Figure 6:**
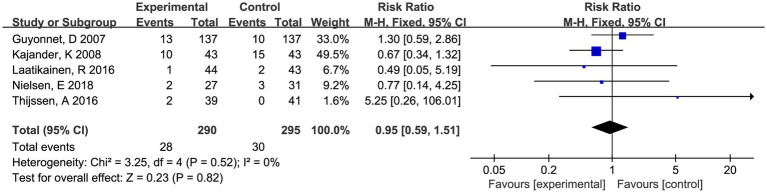
Forest plot of comparison between fermented foods and placebo in term of adverse events. Experimental: fermented food group. Control: placebo group.

Twelve studies depicted the AEs in reports, of which seven studies indicated no treatment-related adverse events during the treatment phase. Five studies presented the data on AEs, and most reported AEs were consistently mild and acceptable. Only two subjects reported serious adverse events, but it was found in control group. Meta-analysis showed that the occurrence rate of AEs was not statistically different from the placebo groups (RR 0.95, 95% CI 0.59–1.51, *p* = 0.82, *I*^2^ = 0%) (Figure 6).

### 3.3 Subgroup analysis

As shown in the Supplementary material 7 (Figure 1), fermented milk had a beneficial effect on symptom relief (RR 1.19, 95% CI 1.01 to 1.39, *p* = 0.04, *I*^2^ = 0%). No other significant results were found in the remaining subgroup analysis.

## 4 Discussion

This systematic review and meta-analysis indicated that patients receiving FFs experienced slightly better improvements in global symptoms of IBS than those treated with placebo. However, subgroup analysis found that only fermented milk had a beneficial effect on symptom relief. In terms of abdominal pain and bloating, we found the score did not significantly change after intervention by FFs compared with placebo.

FFs represent a promising reservoir and vehicle of microorganisms and bioactive compounds and are widely consumed around the world, but few dietary guidelines recommend related-products as healthy supplements. A recent authoritative review from *Nature* detailing the potential mechanisms of FFs for gastrointestinal health suggests that FFs may positively regulate gastrointestinal health through a variety of mechanisms, including fermentation-associated functional compounds, fermentative removal of undesirable compounds and fermentation-associated microorganisms (16). Of those complex mechanisms, intestinal probiotics and low FODMAP diet have been shown to aid bowel symptoms and life quality in IBS patients. For example, a meta-analysis of 82 trials, including more than 10,000 individuals, demonstrated that supplementation with probiotics can improve IBS symptoms (12). Another network meta-analysis found that low FODMAP diet was ranked first for efficacy across all endpoints compared with other alternative dietary advice (17). In our study, we included 16 RCTs involving 6 types of fermented products, of which 13 RCTs stress the probiotic properties of products, and 1 RCT study was associated with FODMAP removal. By quantitative synthesis of those studies, we found that FFs had a statistically significant effect in improving IBS symptoms and this result was light heterogeneity and good robustness.

In 13 RCTs that characterized or labeled as “probiotic foods” which involving single or multi trains of probiotics such as *Lactobacillus*, *Propionibacterium* and *Bifidobacterium*, total 4 types of products were fermented on substrates of milk, rice, oat and sauerkraut, respectively. Indeed, food fermentations are performed on a wider variety of substrates such as meats, fish, fruits, legumes, vegetables, etc., suggesting that the nutritional benefits of FFs have not been fully verified (37). Moreover, 8 RCTs investigated the relationship between fermented milk and IBS in those 13 RCTs, which means the remaining products were small sample sizes. Thus, it was difficult to make a subgroup analysis based on all the products. In our subgroup analysis, we attempted to distinguish the effect of fermented milk and other FFs, and found that only fermented milk had a beneficial effect on IBS symptom relief. This result was mainly influenced by research status: only dairy products and yoghurt were extensively verified in population and approved by dietary guidelines so far (38). By contrast, evidence of non-dairy fermented foods such as kombucha, sauerkraut, kimchi, tempeh, sourdough bread, etc. is mostly limited to chemical analyses or fewer population studies (37).

The beneficial effects of fermented milk mainly derive from the probiotic effect, as evidenced by the fact that the gut microbiota is modified in both animals and humans after administering probiotic fermented milk (39). Indeed, the effect of probiotic administration on IBS has been tested in many clinical trials and meta-analyses. In general, a positive, although modest, effect was noted in those studies (12, 13). With respect to the potential mechanisms, experimental studies have shown that probiotics can influence intestinal permeability, motility, sensitivity, immune system activity, etc. to modify gastrointestinal physiology (40). For example, *Bifidobacterium* alleviates IBS symptoms by normalizing the ratio of anti-inflammatory and proinflammatory cytokine, suggesting an immune-modulating role for the gut (41). Besides the probiotic effect, the compounds during the fermentation of milk also can be regarded as potentially conferring influence on gut health. For example, the fermentation of milk could improve the digestibility of milk proteins and remove lactose, which contributes to the reduction of gastrointestinal symptoms (42). Moreover, the fermentation of milk can lead to biosynthesis and increase the concentrations of various cofactors, such as cobalamin, which can improve intestinal barrier function and villus-to-crypt ratio (43). In the future, as experimental techniques continue to evolve, we anticipate gaining deeper insights into the underlying mechanisms by which fermented milk promotes intestinal health.

The nutritional value of oats can be enhanced through fermentation. Extensive research has shown that fermentation can liberate bioactive compounds from oats (e.g., phenolic compounds and peptides) and reduce the antinutritional compounds (e.g., tannins and phytic acid) through modification of the microstructure (44). The study of a postbiotic fermented oat gruel found a beneficial effect on the colonic mucosal barrier in IBS patients but not on the symptom scoring (22). In our study, we further included two other studies that also showed an insignificant outcome between fermented oat and IBS. However, given the limited sample size and methodological design, we believe that high-quality RCTs specifically designed to investigate fermented oats will be necessary to validate the present findings.

Sauerkraut is a nutritious fermented product, widely consumed around the world, made from cabbage fermented by lactobacillus bacteria with about 3% salt (45). Through culture-dependent techniques, evidence proved that sauerkraut is rich in microbial composition and predominantly contains *Leuconostoc* and *Lactobacillus* spp. (46). Certain *Leuconostoc* and *Lactobacillus* species isolated from sauerkraut showed probiotic potential and antimicrobial activity, preventing the growth of *Salmonella enteritidis* and *Listeria monocytogenes* (47, 48). In addition, sauerkraut contains glucosinolate breakdown products such as kaempferol, which can produce radical scavenging activity to protect from oxidative damage (49). Despite the theoretical benefits, rare studies have been performed to investigate its health value in the real world. Nielsen et al. (30) conducted an RCT to explore the effects of sauerkraut on IBS and the results showed no difference in symptoms between the pasteurized sauerkraut intake group and the unpasteurized sauerkraut intake group. However, because there was no raw cabbage group, the finding may be interfered with by fermentation-derived products and the cabbage itself.

Sourdough starter is generated following the fermentation of wheat by lactic acid bacteria and yeasts. Sourdough bread may confer health benefits by changing the nutritional content of bread in the fermentation process. For example, by specifically designed fermentations, the sourdough process can decrease undesirable compounds such as FODMAP, gluten and phytic acid (16). In our study, 2 RCTs were conducted based on sourdough fermented products (27, 28). Of these, the study of Laatikainen et al. (27) showed significantly milder flatulence, abdominal pain, intestinal cramps, rumbling and total symptoms scores by consumption of a low FODMAP sourdough rye bread, compared to normal rye bread. However, the study of Lluansí et al. (28) showed that the remission rate of IBS symptoms was not significantly different between sourdough bread and modern bread. Regrettably, due to discrepancies in trial design, particularly regarding the selection of control groups, we concurred that it is inappropriate to combine these two studies as a subgroup to explore the health benefits of sourdough fermentation for IBS.

Kombucha is a fermented tea beverage enriched with phenolic compounds like catechins, theaflavins, and thearubigins (50). Polyphenols have been reported to possess antioxidant activity and induce the growth of beneficial gut microorganisms *in vivo* and *in vitro* (51). We found only one study that explored the effect of Kombucha on the management of IBS in the process of literature retrieval. The study included 40 IBS-C female patients and showed that Kombucha significantly improved the symptom of incomplete bowel emptying and increased the stool frequency (24). However, given the study limitations like short duration (only 10 days) and small sample size, these findings require additional validation through larger-scale RCTs.

It is crucial for consumers to have assurance that their food is safe. Although FFs are generally considered safe, some factors (e.g., inappropriate materials, unhygienic environments, and non-compliance with product processes) result in the introduction of health hazards such as mycotoxins and plant toxins into production process (52). The microbial metabolites of some FFs may also induce safety risks, such as histamine (formed by lactic acid bacteria in protein fermentation), which can cause mild to more severe effects (53). In our study, we found FFs to be safe in patients with IBS and no serious adverse events occurred with the use of FFs. It is worth noting that the included FFs have expanded to industrial levels, but the general types of fermented products are still produced on a community scale or household scale, with variable levels of hygiene. A recent review pointed out that the current regulatory guidelines are not mature enough to adequately regulate the increasing FFs (54). Thus, specific regulations with rigorous safety testing for specific FFs are needed to outline specifications for composition, safety, communication, and distribution. Simultaneously, food research institutes should advance the safe fermentation technologies to ensure products with good flavor, mouthfeel, and health-related attributes.

To our knowledge, this was the first comprehensive systematic review and meta-analysis to systematically compile human interventional evidence to assess the efficacy of FFs on IBS patients. Those evidences were important for providing justification to incorporate FFs as a recommended category in dietary guidelines. To ensure the comprehensiveness of the literature search, we conducted an extensive review of pertinent literature and developed comprehensive search strategies. All the steps were conducted following standard methods and pre-defined protocol. Nevertheless, significant limitations arise from the nature of the studies available for synthesis. Firstly, except for fermented milk, the RCTs of the remaining products were rare and relatively small samples as well as some results were insignificant and inconsistency. Thus, we could be criticized because we were unable to draw a definitive conclusion and perform a quality subgroup analysis about specific products. Secondly, the design of RCTs lacked uniformity in endpoint, duration, and IBS subtype in quite a few studies, which means that we need to cautiously interpret outcomes even in mild heterogeneity. We analyzed four distinct endpoints with reference to previous IBS meta-analyses (12) and discovered significant results in two of them, while there was no difference in the two outcomes of abdominal pain and abdominal distension. This result could be due to patients’ different baseline severities, placebo effects, etc. For instance, we noticed that in most studies, the abdominal pain score was mild to moderate, which might be hard to improve completely. However, it is possible for patients to have an improvement in the frequency of abdominal pain. It may partly account for why we observed positive outcomes in overall symptoms but not in single symptoms. In addition, we frequently observed a high risk of attrition bias and reporting bias. Even if we used an ITT analysis and attempted to acquire original data, the evidence from those with a high risk of bias needs to be carefully evaluated. Larger RCTs with greater methodological rigor should be designed to further verify our findings. New RCTs should be designed to account for FF categories, cohort size, placebo choice, intervention durations, covariates, and especially dietary recall variables, etc. In addition, collecting information from large population-based diet and health databases (such as the NHANES database) would facilitate understanding the potential values of FFs (55).

## 5 Conclusion

In summary, fermented foods, especially fermented milk with probiotics properties, may serve as a viable alternative therapy for irritable bowel syndrome. However, given the status of rare study numbers, relatively small samples and moderate certainty evidence quality, this conclusion should be interpreted with caution. Moreover, the applicability of these findings should be narrowly defined until further research is conducted.

## Data Availability

The original contributions presented in the study are included in the article/Supplementary material, further inquiries can be directed to the corresponding author.
