# Supplementary material for: Efficacy of fermented foods in irritable bowel syndrome: a systematic review and meta-analysis of randomized controlled trials
Source: Front Nutr. 2025 Jan 7;11:1494118. doi: 10.3389/fnut.2024.1494118 (PMC11747498; doi:10.3389/fnut.2024.1494118)
Supplement: Supplementary file 1 [file Table_1.DOCX]

Supplement 1. Search strategy for RCTs investigating the effect of fermented foods on irritable bowel syndrome.

("IBS" OR "irritable bowel syndrome" OR "spastic colon" OR "irritable colon" OR "colon" OR " functional adj5 bowel") AND ("Fermentation" OR "Fermented food" OR "Yoghurt" OR "Yogourt" OR "Yoghourt" OR "Yogurt" OR "Soured milk" OR "Buttermilk" OR "Acidophilus-milk" OR "Fermented milk" OR "Amasi" OR "Labneh" OR "Dahi" OR "Lassi" OR "Clabber" OR "Amasi" OR "Curd" OR "Filmjolk" OR "Viili" OR "Cheese" OR "Raw cheese" OR "Cheddar cheese" OR "Stilton cheese" OR "Sour cream" OR "Koumiss" OR "Kumys" OR "Kefir" OR "Sourdough" OR “Sourdough bread” OR “Kombucha” OR "tofu" OR "bean curd" OR "natto" OR "tempeh" OR "soy cheese" OR "soy food" OR "soy sauce" OR "soy milk" OR "soy beverage" OR "Doubanjiang" OR "Douchi" OR "Tianmianjiang" OR "Chungkookjang" OR "Doenjang" OR "Miso" OR "Jiang" OR "Kimchi" OR "Sauerkraut" OR "Gochujang" OR "Pickle" OR "Tsukemono" OR "Garri" OR "Gochujang" OR "Suan cai" OR "Fish sauce" OR "Cincalok" OR "Surstromming" OR "Katsuobushi" OR "Ham" OR "Prosciutto" OR "Sausage" OR "Teewurst" OR "Thuringer" OR "Lebanon bologna" OR "Salaami" OR "Pepperoni" OR "Bread" OR "Ogi" OR "Dosa" OR "Kvass") AND (intervention[tiab] OR RCT[tiab] OR controlled trial[tiab] OR randomized[tiab] OR random[tiab] OR Randomly[tiab] OR Placebo[tiab] OR Assignment[tiab] OR clinical trial[tiab] OR trial[tiab] OR randomised[tiab] OR Randomized Controlled Trial[Publication Type] OR Controlled Clinical Trial[Publication Type] OR Clinical Trial[Publication Type])

Supplement 2. Risk of bias graph and summary


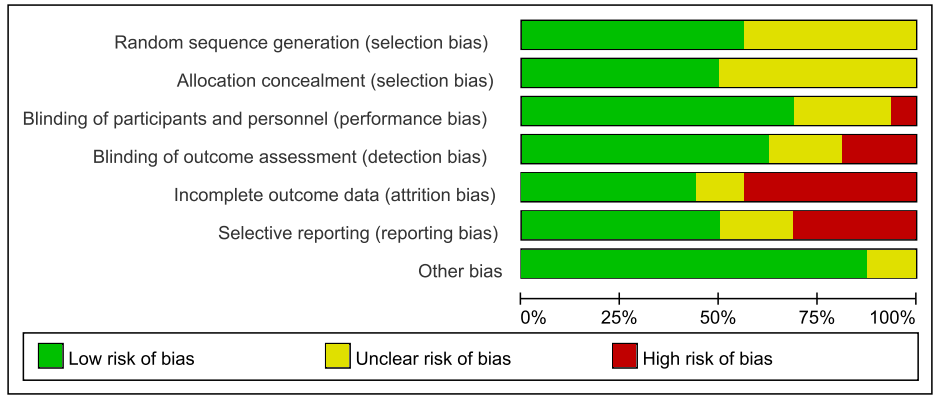


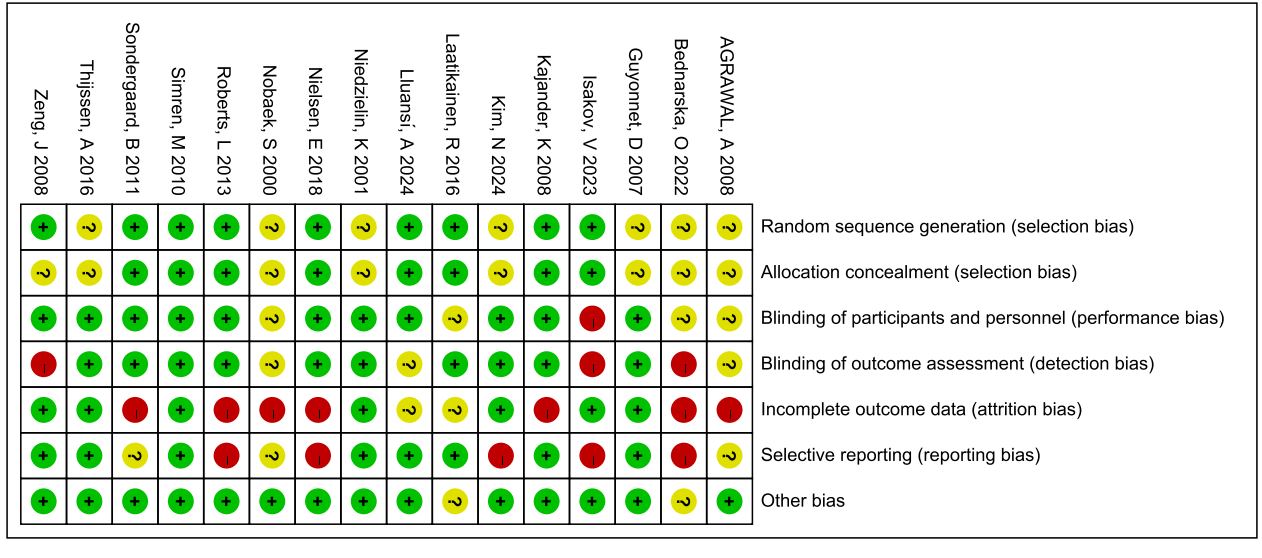


Supplement 3. Funnel graph


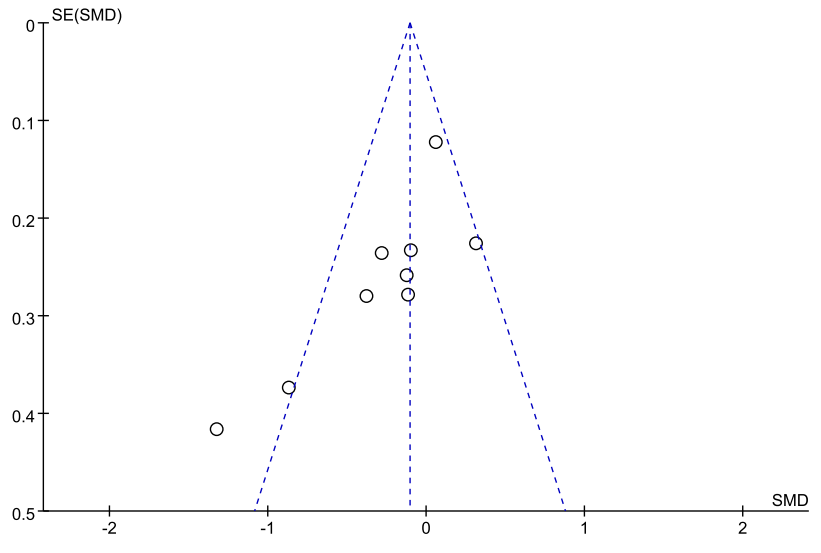


Fig 1. Funnel graph of comparison between fermented foods and placebo in term of symptom relief.


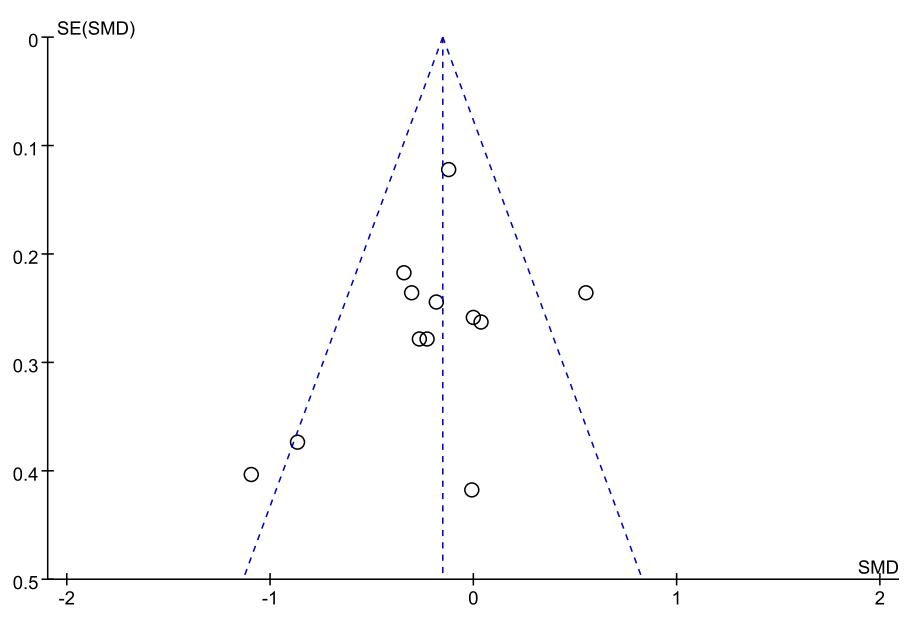


Fig 2. Funnel graph of comparison between fermented foods and placebo in term of global symptom score.


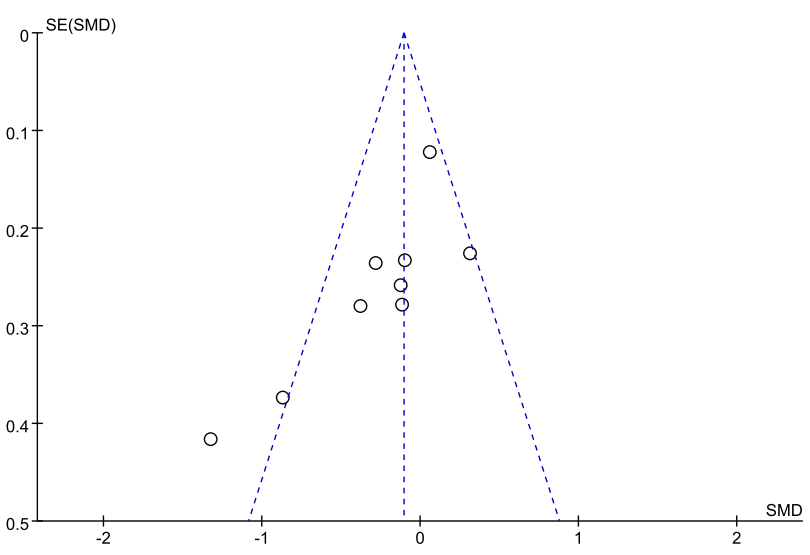


Fig 3. Funnel graph of comparison between fermented foods and placebo in term of abdominal pain score.


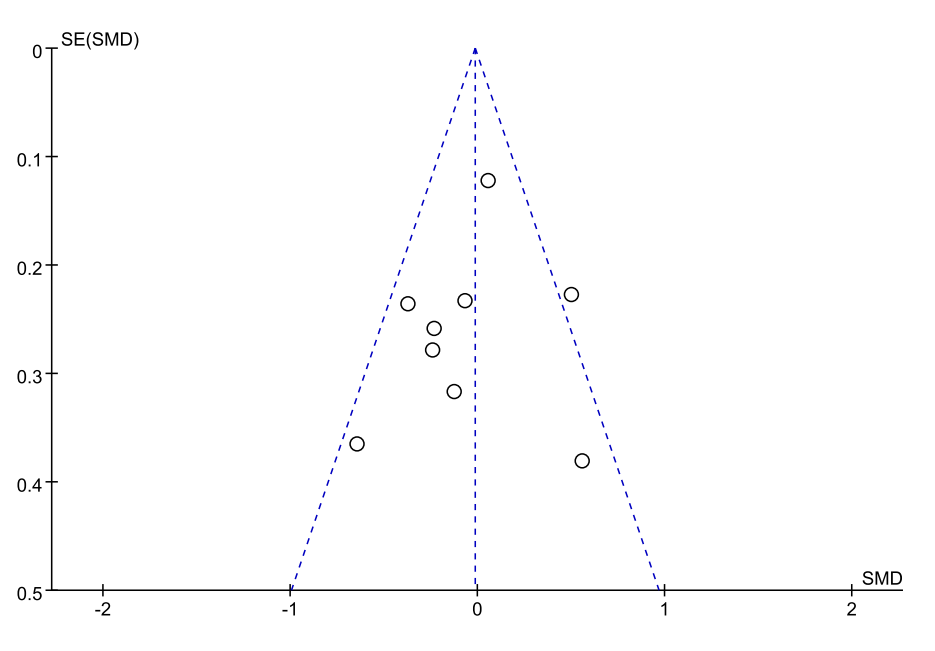


Fig 4. Funnel graph of comparison between fermented foods and placebo in term of abdominal bloating score.

Supplement 4. Begg and Egger test


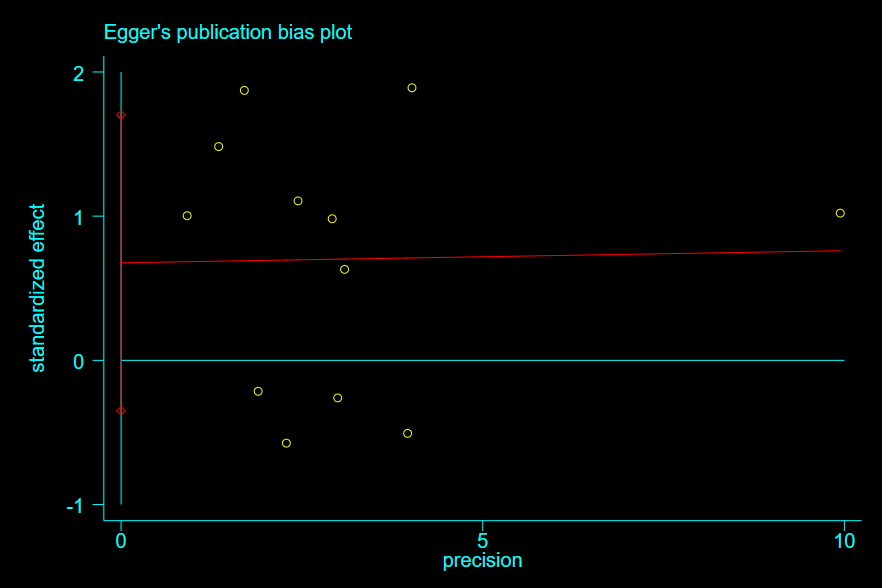

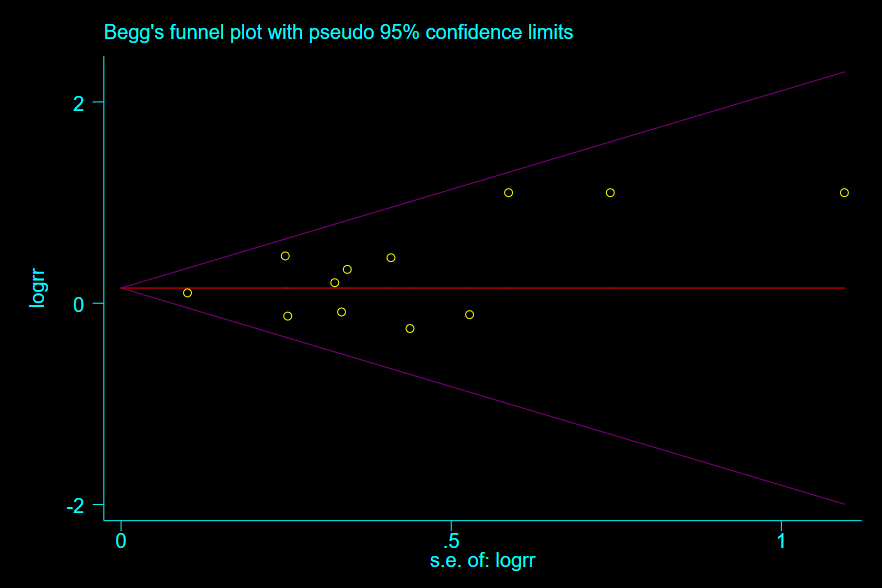


Fig 1. Begg and Egger test of comparison between fermented foods and placebo in term of symptom relief.


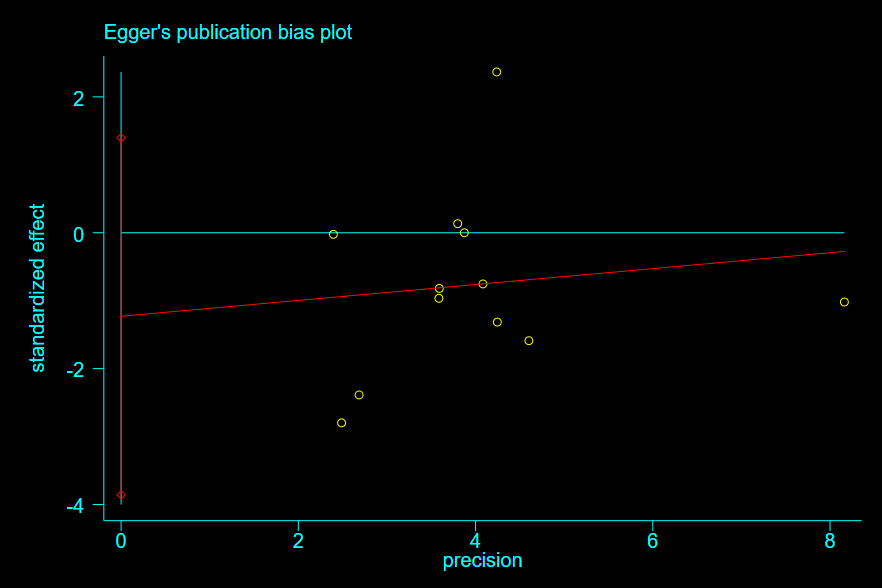


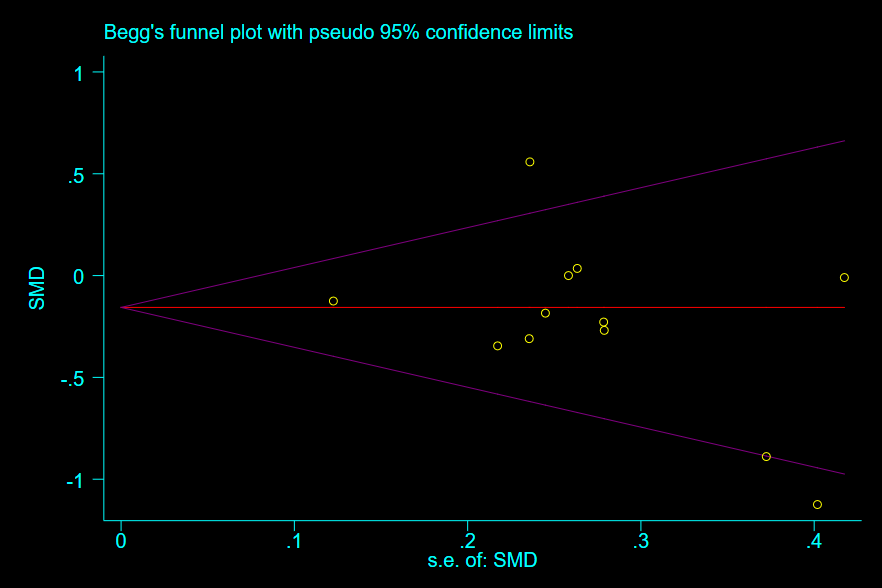


Fig 2. Begg and Egger test of comparison between fermented foods and placebo in term of global symptom score.


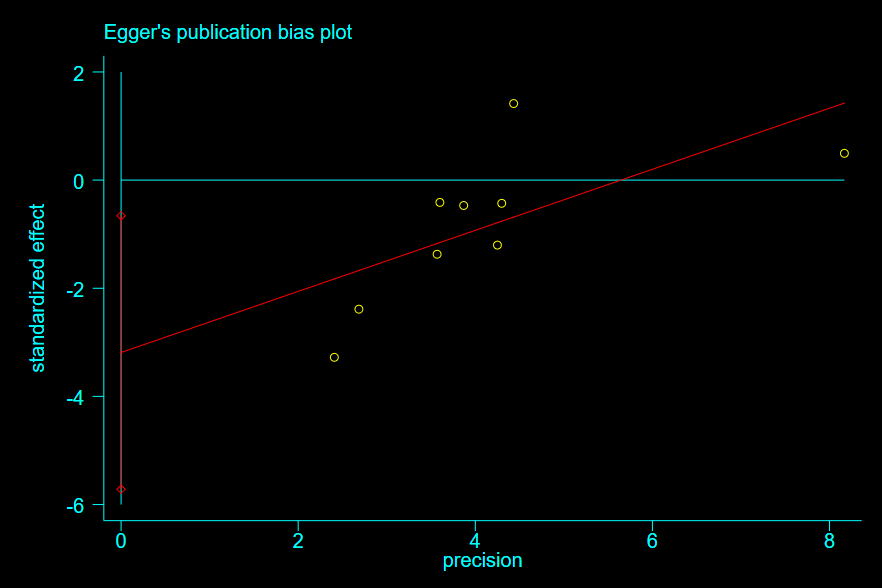


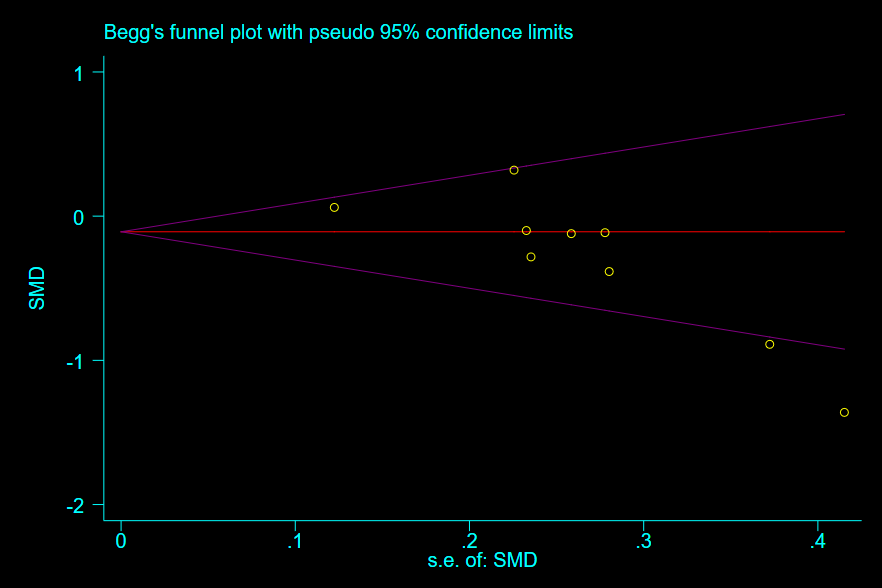


Fig 3. Begg and Egger test of comparison between fermented foods and placebo in term of abdominal pain score.


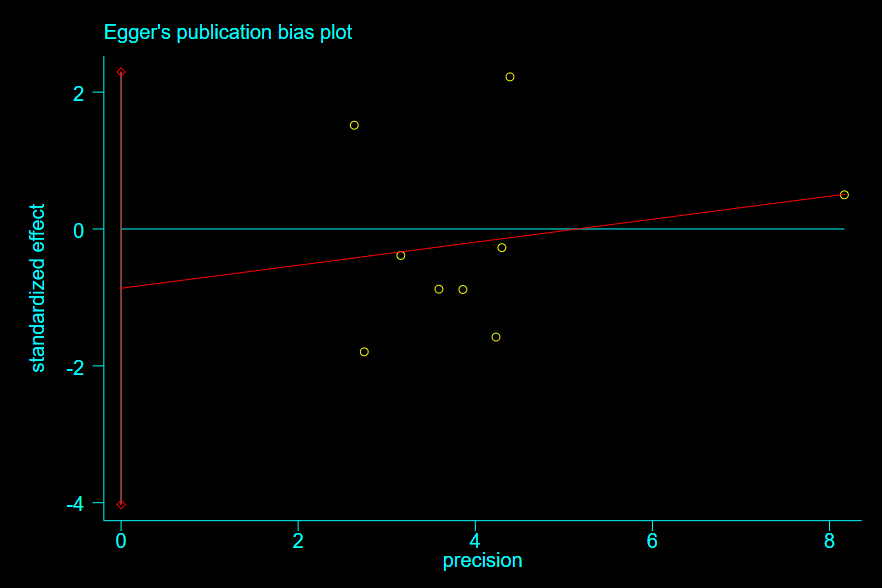

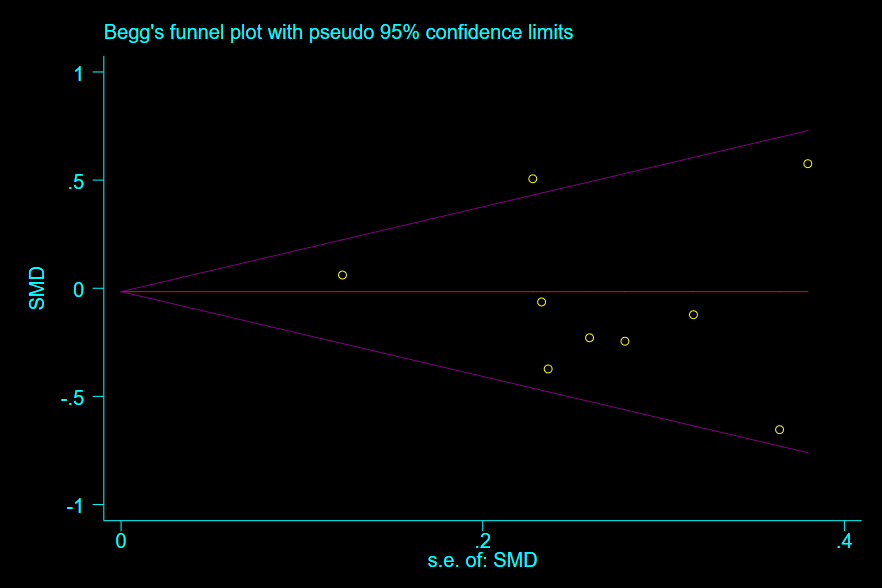


Fig 4. Begg and Egger test of comparison between fermented foods and placebo in term of abdominal bloating score.

Supplement 5. Sensitivity analysis of comparison between fermented foods and placebo in term of symptom relief.


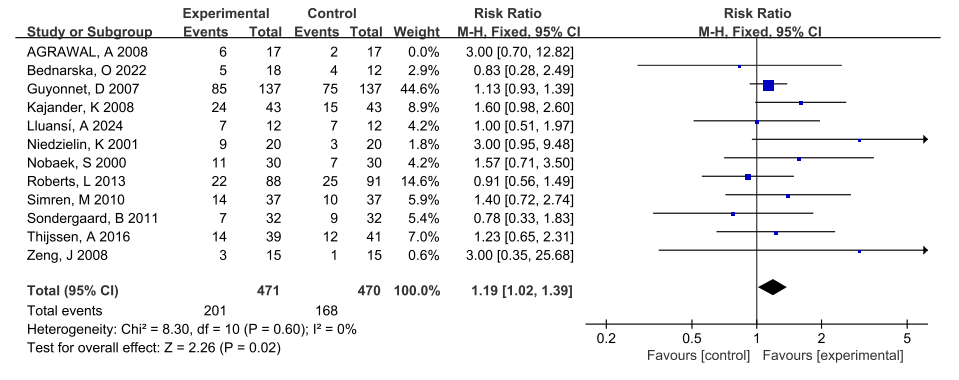


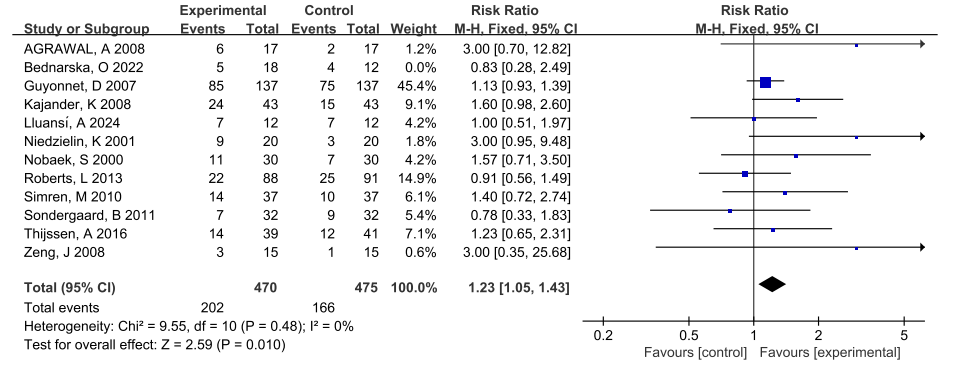


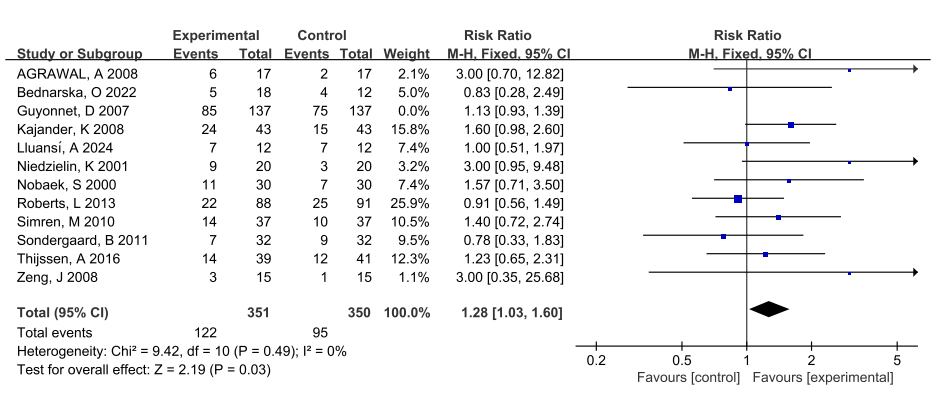


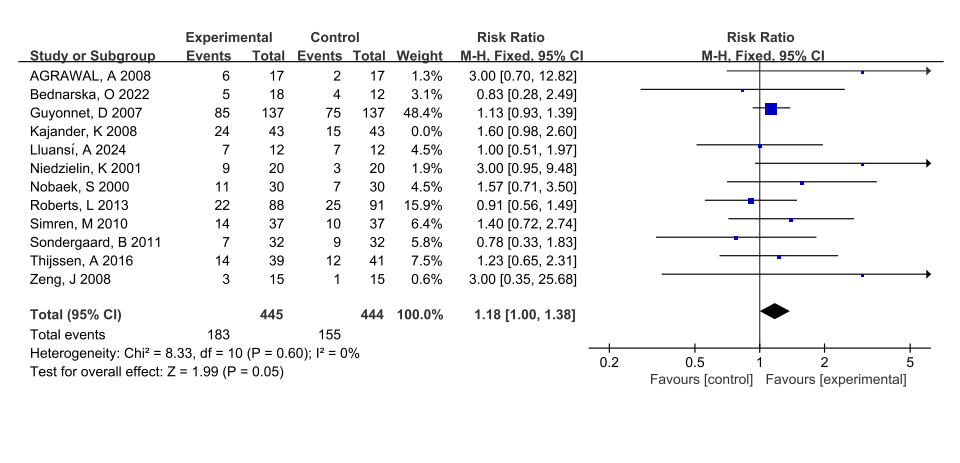


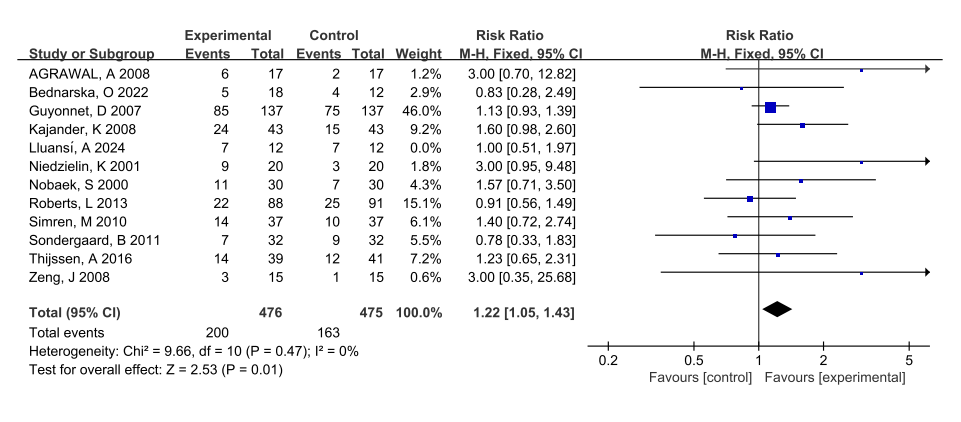


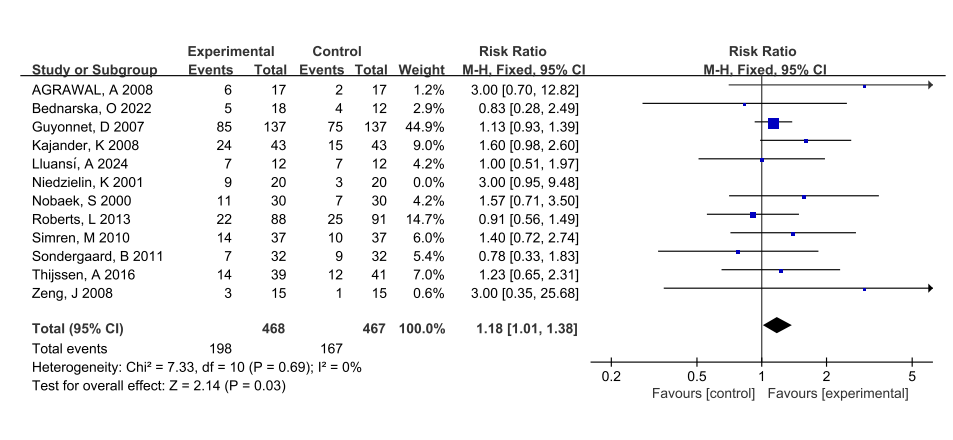


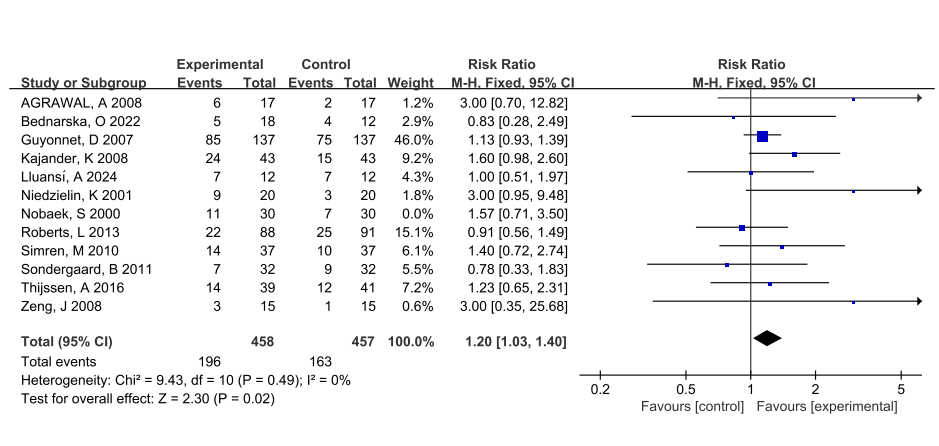


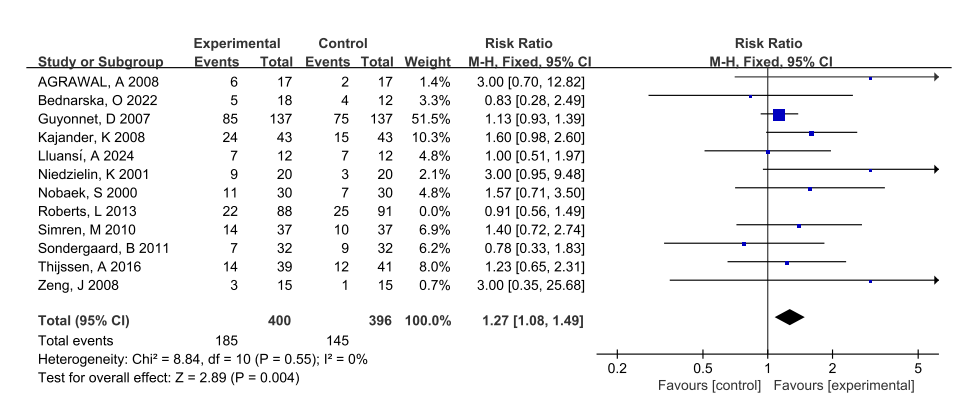


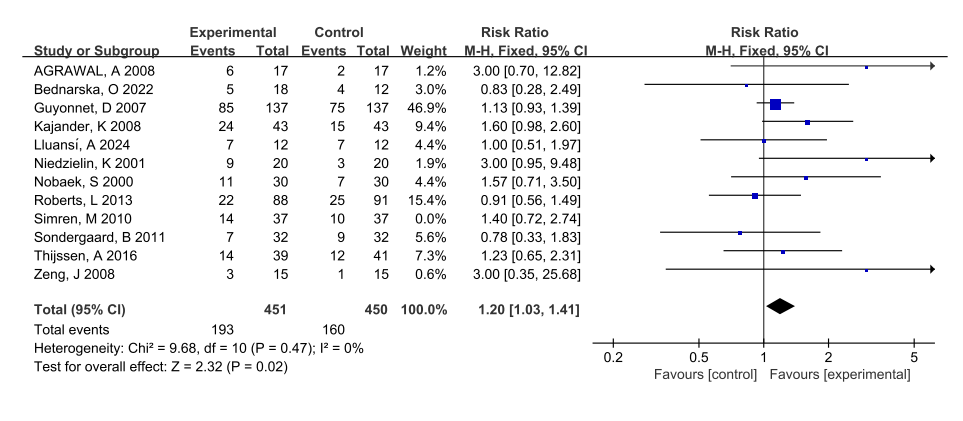


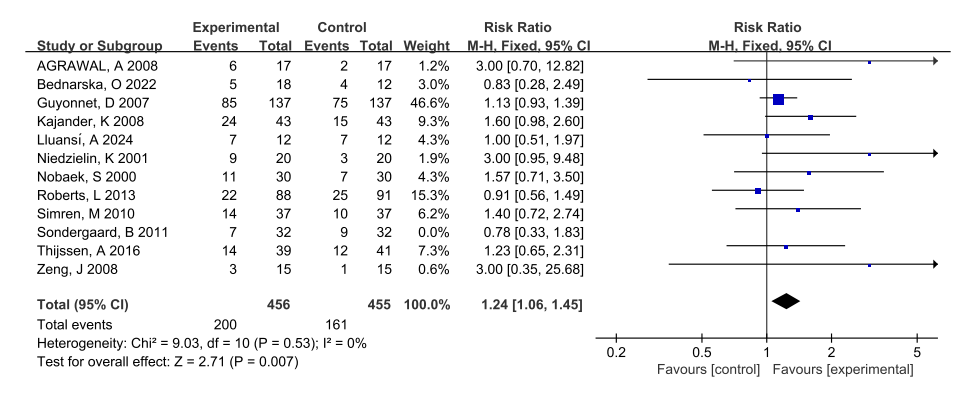


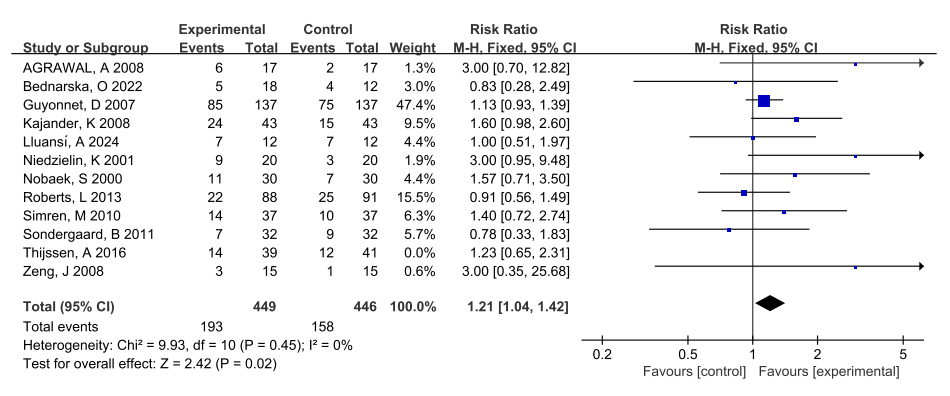


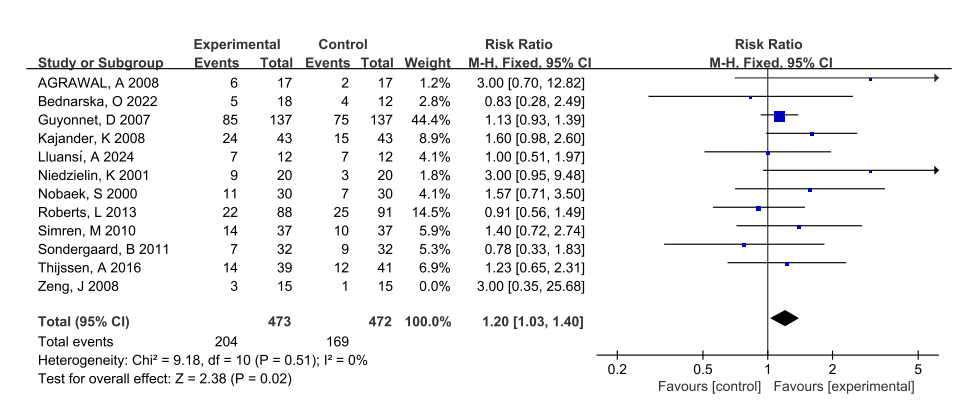


Experimental: fermented food group. Control: placebo group

Supplement 6. Sensitivity analysis of comparison between fermented foods and placebo in term of global symptom score.


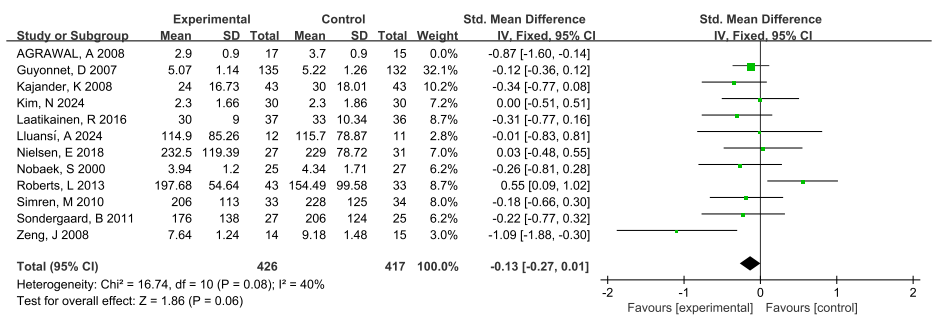


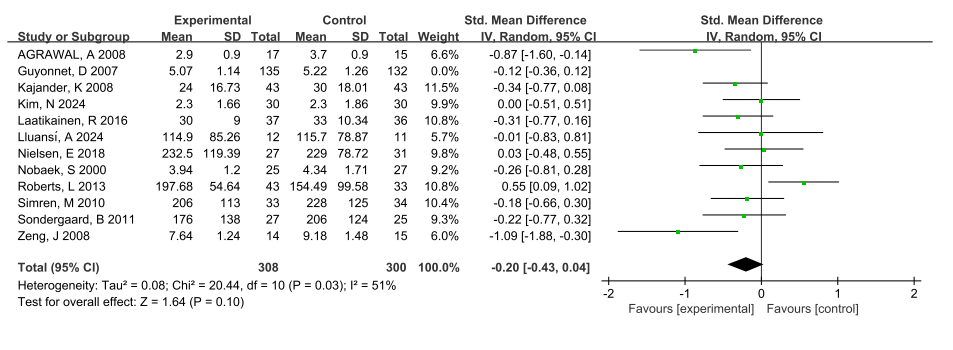


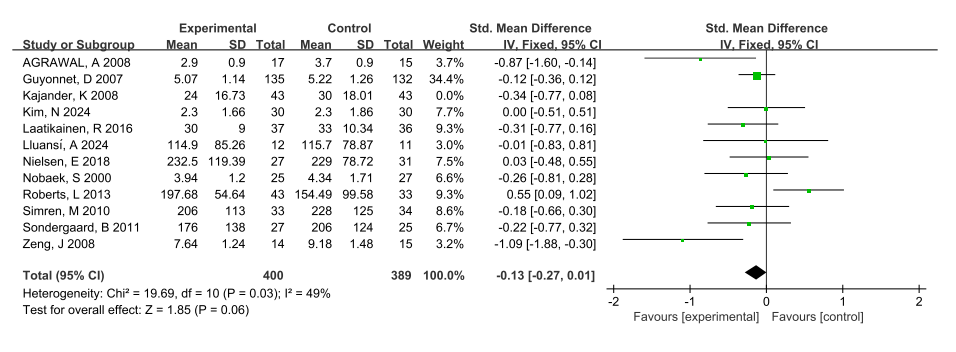


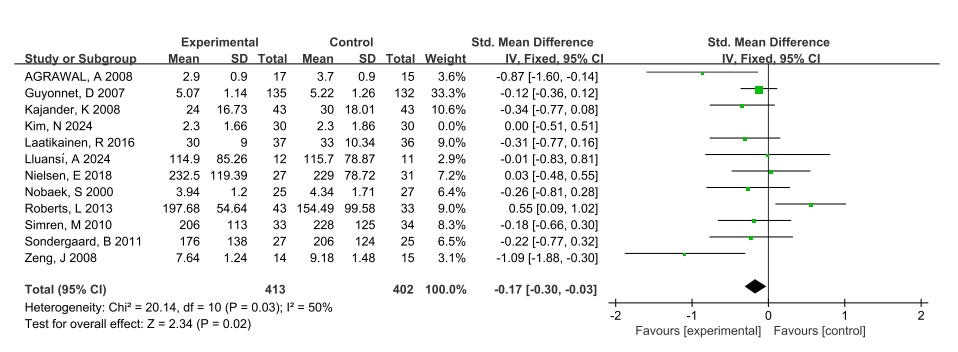


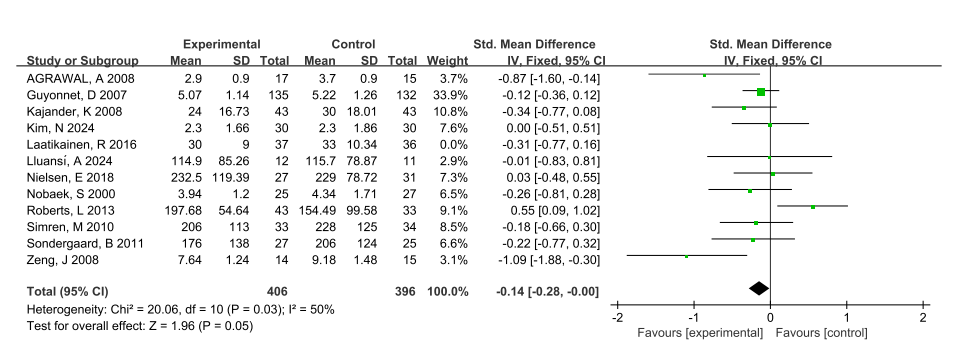


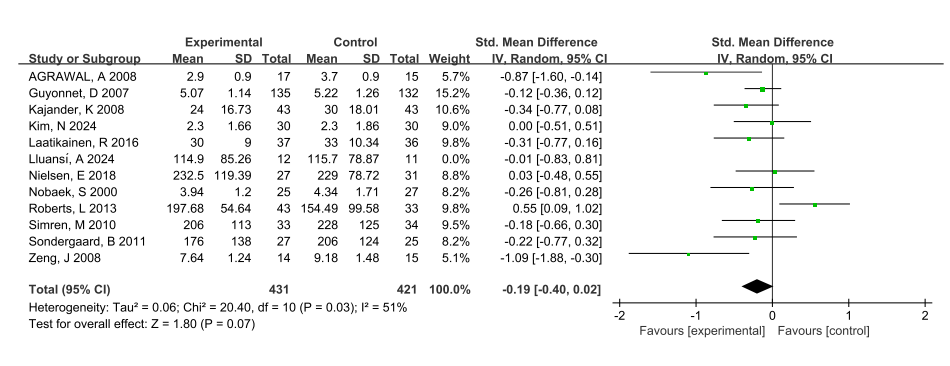


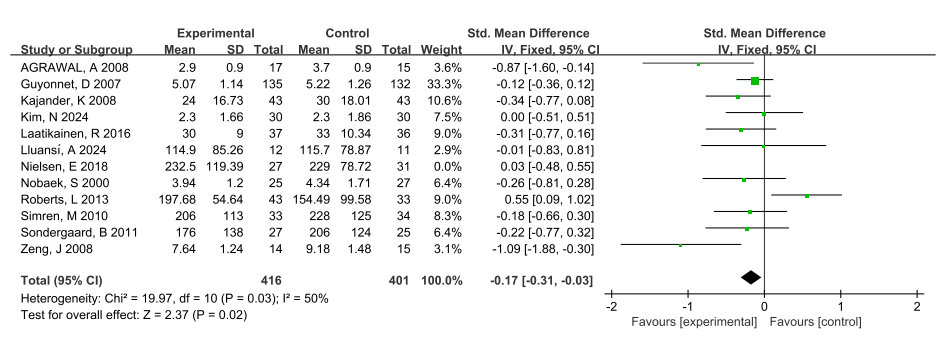


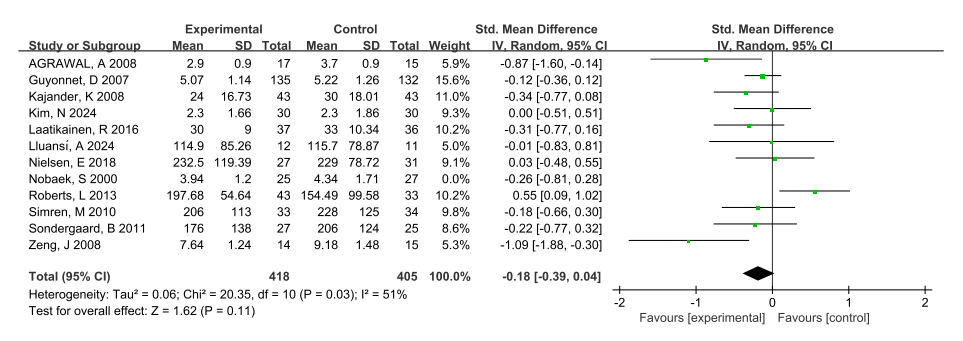


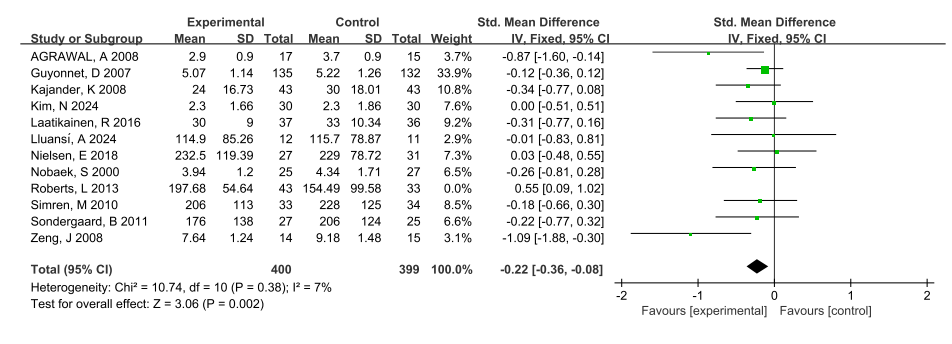


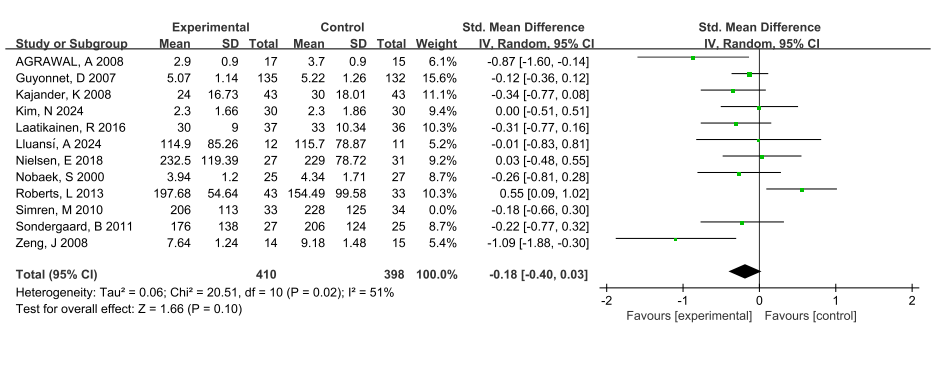

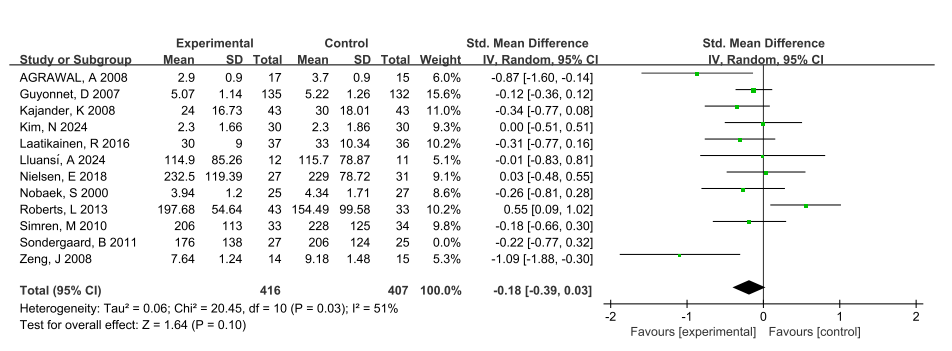


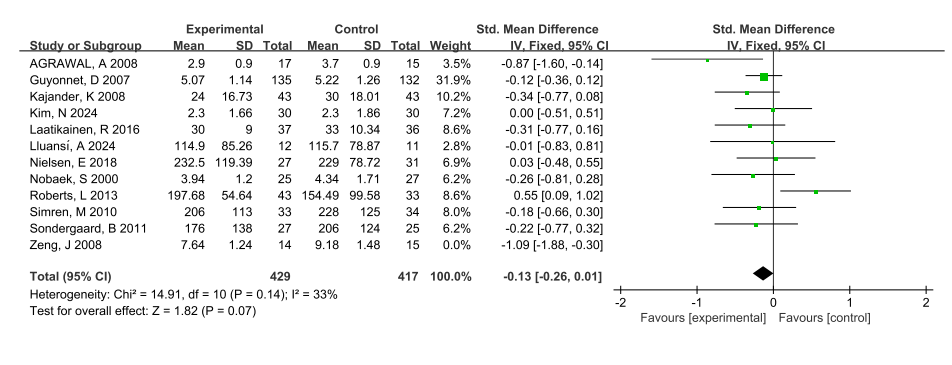


Experimental: fermented food group. Control: placebo group

Supplement 7. Subgroup analysis


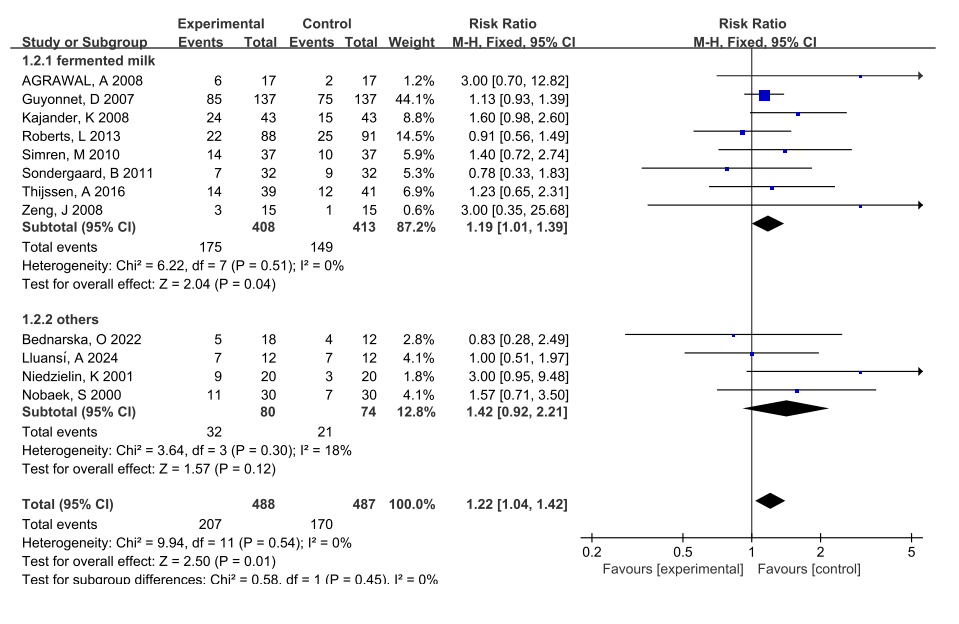


Fig 1. Subgroup analysis of comparison between fermented foods and placebo in term of symptom relief.


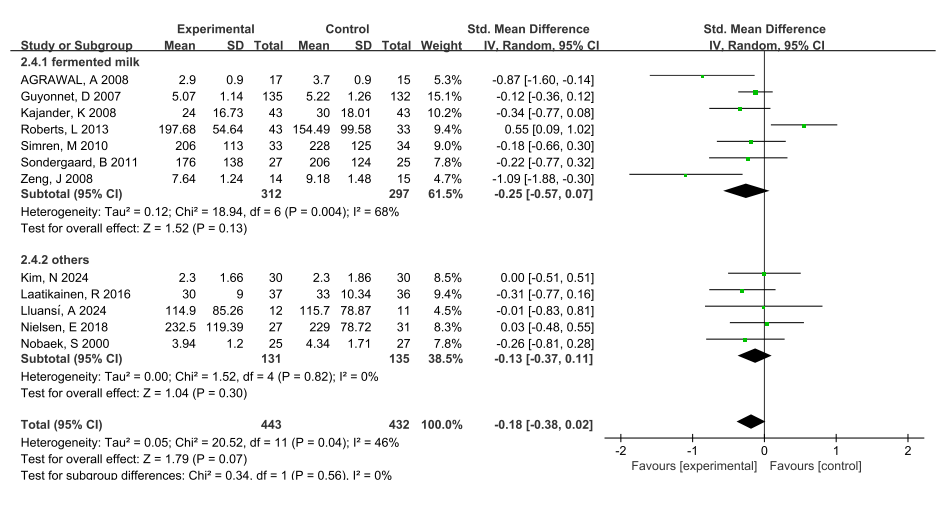


Fig 2. Subgroup analysis of comparison between fermented foods and placebo in term of global symptom score.


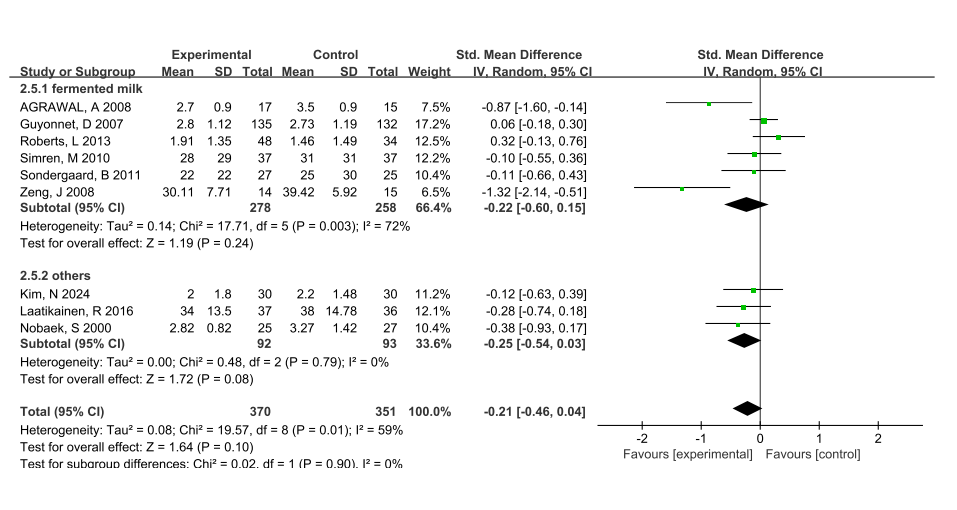


Fig 3. Subgroup analysis of comparison between fermented foods and placebo in term of abdominal pain score.


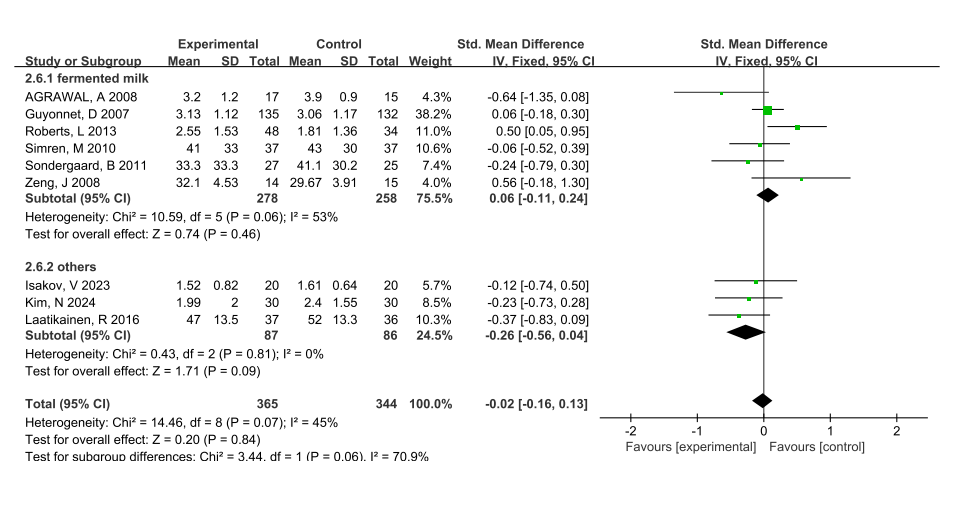


Fig 4. Subgroup analysis of comparison between fermented foods and placebo in term of abdominal bloating score.


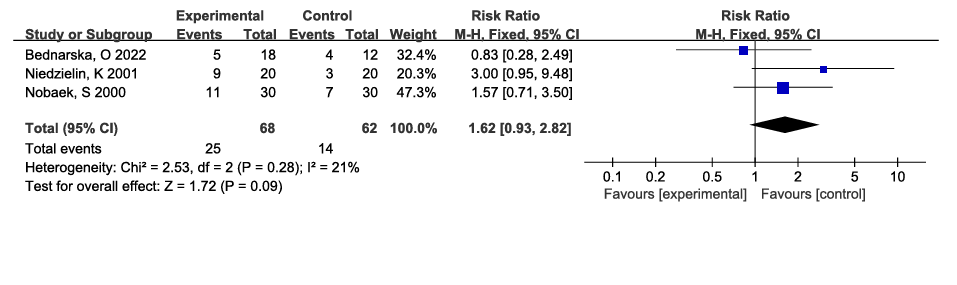


Fig 5. Subgroup analysis of comparison between fermented oat and placebo in term of symptom relief.

Experimental: fermented food group. Control: placebo group
